# Survey of antiobesity drug prescribing for obese children and young people in UK primary care

**DOI:** 10.1136/bmjpo-2017-000104

**Published:** 2017-10-25

**Authors:** Billy White, Yingfen Hsia, Sanjay Kinra, Sonia Saxena, Deborah Christie, Russell M Viner, Ian C K Wong

**Affiliations:** 1 Department of Population Health Sciences, UCL Institute of Child Health, London, UK; 2 Department of Adolescent Medicine, University College London Hospital, London, UK; 3 Paediatric Infectious Diseases Research Group, St George’s University of London, London, UK; 4 Department of Non-communicable Disease Epidemiology, London School of Hygiene & Tropical Medicine, London, UK; 5 Department of Primary Care and Public Health, Imperial College London, London, UK; 6 Department of Child and Adolescent Psychological Services, University College London Hospital, London, UK; 7 Department of Epidemiology and Public Health, UCL, London, UK; 8 Centre for Paediatric Pharmacy Research, UCL School of Pharmacy, London, UK

**Keywords:** obesity, adolescent health

## Abstract

**Objectives:**

Antiobesity drug (AOD) prescribing in children and young people (CYP) in primary care is rising with high rates of discontinuation. Little is known about prescribing in this group in terms of patient demographics and comorbidities, reasons for initiation and discontinuation, or adherence to national guidelines.

**Design:**

Questionnaire survey to general practitioners (GPs) identified using a nationally representative primary care database covering 6% of UK population.

**Setting:**

UK-wide primary care.

**Participants:**

Patients were eligible if prescribed an AOD aged ≤18 years between 2010 and 2012. A total of 151 patients from 108 unique practices were identified via national prescribing database, with responses for 119 patients (79%) from 84 practices; 94 of 119 (79%) were eligible for inclusion.

**Primary and secondary outcomes:**

Survey of GP prescribing habits of AODs to CYP. We audited orlistat usage against the National Institute for Health and Care Excellence (NICE) guidance.

**Results:**

47% were prescribed metformin, 59% orlistat and 5% both drugs. Orlistat was largely prescribed by GPs independently (49/55 prescriptions, 89%) and metformin by GPs on specialist recommendation (12/44, 27%). Orlistat was largely prescribed in those over 16 years of age without physical comorbidities. Metformin was initiated for treatment of polycystic ovarian syndrome (70%), insulin resistance (25%) and impaired glucose control (9%). Median supply of metformin was 10.5 months (IQR 4–18.5 months) and 2.0 months (1.0–4.0) for orlistat (p≤0.001). Drug terminations were largely due to families not requesting repeat prescriptions. NICE guidance adherence was low; 17% of orlistat prescriptions were initiated by specialists, and 56% had evidence of obesity-related comorbidity. GPs reported lower confidence in prescribing AOD to CYP compared with adults (10-point Likert score median 3 vs 8, p<0.001).

**Conclusions:**

Prescribing of AOD in primary care is challenging with low adherence to NICE guidance. Further work is needed to better support GPs in the use of AOD in CYP.

**What is already known on this topic?**

- Orlistat and metformin are both used as antiobesity drugs (AOD) in children and young people, yet only orlistat is approved in this age group.
- UK primary care prescribing data show increasing use and high levels of drug discontinuation, with half of orlistat prescriptions not being continued beyond 1 month.
- One qualitative study eshowed frequent cessation by families independent of their doctors, usually because the perceived advantages did not outweigh the medication side effects.

**What this study hopes to add?**

- Orlistat was largely prescribed independently by general practitioners to patients aged 16 years and over without physical comorbidities.
- Metformin was largely initiated by specialists for subjects with comorbidities, including polycystic ovarian syndrome, insulin resistance and impaired glucose tolerance.
- Adherence to the National Institute for Health and Care Excellence guidelines for orlistat prescribing to children and young people was low.

## Introduction

Little is known about use of medication for obesity in children and adolescents in the UK, particularly use in primary care. Orlistat is currently the only licensed antiobesity drug (AOD) in the UK since sibutramine was withdrawn due to concerns about cardiovascular safety.^1 2^ However, the most commonly used drug for obesity in children and young people (CYP) is metformin, an antidiabetes drug used off-licence to treat the metabolic sequelae of obesity in CYP, although not formally classed as an AOD.^3 4^ Both orlistat and metformin appear to offer small benefits for body mass index (BMI) loss in CYP; systematic reviews show small reductions in BMI compared with placebo, orlistat by 0.83 kg/m^2^^5^ and metformin by 1.4 kg/m^2^ (at 6–12 months and 6 months, respectively).^6^

In the UK, the National Institute for Health and Care Excellence (NICE) guidance recommends community-based lifestyle modification programmes as the first tier of weight management for childhood obesity, with pharmacotherapy as a second-line treatment.^2^ Their guidance, summarised in box 1, only covers use of orlistat, which they state should be prescribed only in exceptional circumstances for those with obesity-related comorbidities (life-threatening in those under 12 years of age) and only prescribed by teams with expertise in these conditions.

Box 1 **Summary of 2014National Institute for Health and Care Excellence guidance for prescribing of orlistat to children and young people**

1.8.4—Drug treatment is not generally recommended for children younger than 12 years.

1.8.5—In children younger than 12 years, drug treatment may be used only in exceptional circumstances, if severe comorbidities are present. Prescribing should be started and monitored only in specialist paediatric settings.

1.8.6—In children aged 12 years and older, treatment with orlistat is recommended only if physical comorbidities (such as orthopaedic problems or sleep apnoea) or severe psychological comorbidities are present. Treatment should be started in a specialist paediatric setting, by multidisciplinary teams with experience of prescribing in this age group.

1.8.7—Do not give orlistat to children for obesity unless prescribed by a multidisciplinary team with expertise in drug monitoring, psychological support, behavioural interventions, interventions to increase physical activity and interventions to improve diet.

1.8.8—Drug treatment may be continued in primary care, for example, with a shared care protocol if local circumstances and/or licensing allow.

1.9.2—Adults and children: If there is concern about micronutrient intake adequacy, a supplement providing the reference nutrient intake for all vitamins and minerals should be considered, particularly for vulnerable groups such as older people (who may be at risk of malnutrition) and young people (who need vitamins and minerals for growth and development).

1.9.11—If orlistat is prescribed for children, a 6-month to 12-month trial is recommended, with regular review to assess effectiveness, adverse effects and adherence.

Randomised trial data on orlistat and metformin come from specialist clinical settings and largely from outside the UK. Very little is known about how these AODs are prescribed and used in actual practice. Pharmacoepidemiology studies of AOD prescribing in primary care in the UK show increasing use of AODs, but also high levels of drug discontinuation, with approximately half the prescriptions of orlistat not being continued beyond 1 month.^5^ The one qualitative study examining adolescent use of AOD showed frequent cessation by families independent of their doctors, usually because the perceived advantages did not outweigh the medication side effects that they endured with often minimal professional support.^7^ These data suggest that the effectiveness of AOD in ‘real life’ settings may be considerably less than shown in trials, and suggest a need to identify strategies to improve the effectiveness of AODs for CYP.

We undertook a questionnaire survey of general practitioners (GPs) prescribing AODs to CYP to better understand their use in primary care in the UK. We sought to characterise patient demographics, quantify adherence to NICE guidance and identify primary care perceptions of AOD with the long-term aim of optimising AOD prescribing and efficacy.

## Materials and methods

We used routinely collected primary care data from The Health Improvement Network (THIN) database to identify CYP aged up to and including 18 years prescribed orlistat or metformin between 31 May 2010 and 31 May 2012. We excluded patients prescribed metformin for type 2 diabetes.

THIN covers approximately 6% of the UK population, with 3.6 million active patients from 587 general practices using the Vision General Practice System.^8^ These practices are broadly representative of practices in the UK in respect of patients’ demographics and characteristics.^9^ Questionnaire administration was undertaken by THIN Additional Information Services (THIN AIS), an independent research organisation affiliated with THIN, with data protection firewalls.

A paper questionnaire was sent to the GP practices of all identified CYP to collect patient-level data (see online supplementary appendix file 1 for full questionnaire). The questionnaire was designed by two paediatricians, an academic GP, a psychologist, a pharmacist and a GP representative following recommendations for good practice in survey research.^10^ GPs were contacted up to three times over 3 months until the questionnaire was returned. GPs received a £35 payment for each completed and returned questionnaire. THIN AIS anonymised questionnaires prior to analysis by the study team.

10.1136/bmjpo-2017-000104.supp1 Supplementary file 1

Year of birth, practice ID and region were provided by THIN AIS. All other data were provided by GPs using existing medical records, including ethnicity. We assumed AOD termination if no prescription had been issued within 3 months of the survey. Age at first prescription was calculated from the midpoint of birth year, as month and day of birth were not provided due to data protection restrictions.

BMI and zBMI were calculated from GP-derived height and weight measurements using the LMS method and UK reference data.^11^ We audited orlistat use against NICE 2006 recommendations, which remain unchanged in the 2014 update, bar some text clarifications.^2 12^ For audits against NICE criteria only, we assumed birth date of 1 January to ensure that no subjects were misclassified as children if they were 18 years of age. Questionnaire responses were read by two researchers and any differences agreed.

### Analyses

Analyses were conducted using STATA V.11.0. Simple descriptive statistics were used for the majority of data. Duration of drug use was compared using Wilcoxon-Mann-Whitney test (highly skewed data) and paired Likert scores using Wilcoxon signed-rank test.

Handwritten free-text comments were read and coded using a general thematic coding methodology.^13^ Models were developed through an iterative process, in which the initial model was reviewed using constant comparison techniques (in which successive items of data are appraised and compared to ensure the code is reflective of all) and the models revised accordingly.

## Results

### Patient demographics

**Figure 1:**
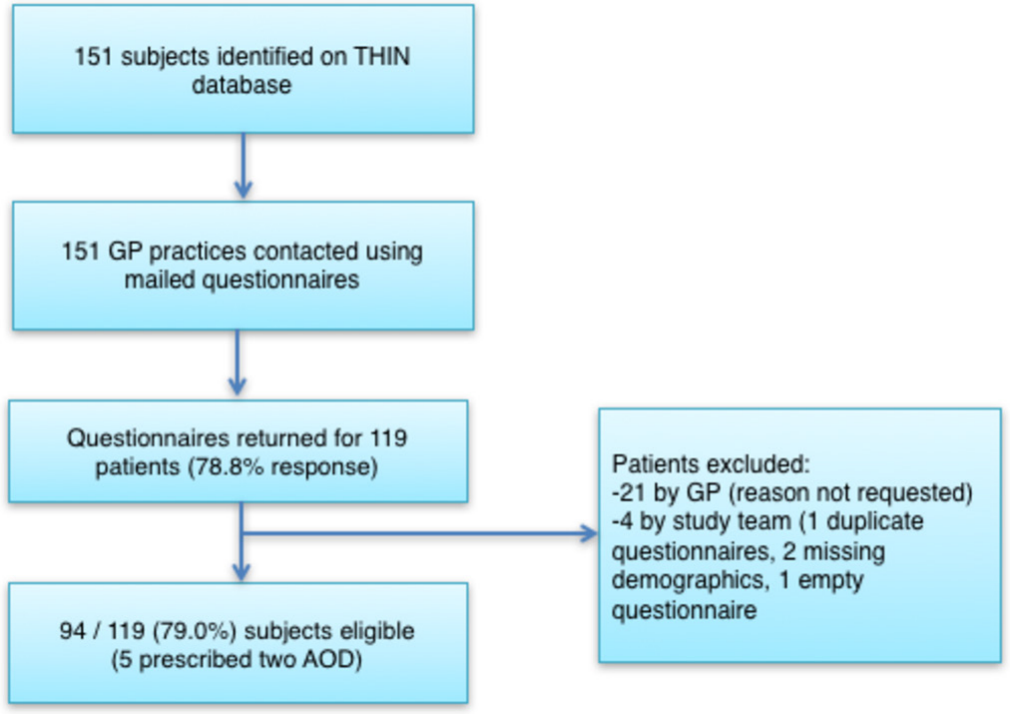
Patient sampling. THIN = The Health Improvement Network, GP = general practitioner, AOD = anti-obesity drug.

Figure 1 summarises patient sampling; 151 patients were identified on THIN database from 108 unique GP practices, with 79% GP response rate (119 of 151 identified patients) from 84 unique practices. A total of 94 subjects were eligible (86% female, 45% British, 31% white/Caucasian, 4% Asian, 4% other, 16% unknown ethnicity). The majority came from England (79%), with the remaining from Wales (12%), Scotland (7%) and Northern Ireland (2%).

A total of 99 AOD initiations occurred in 94 subjects (five subjects were prescribed both orlistat and metformin), consisting of 44 metformin (47% of sample) and 55 orlistat (59%) prescriptions. Drugs were initiated in 68 practices, with 46 practices prescribing one drug each, 15 practices two drugs each, 6 practices three drugs each and 1 practice prescribing five drugs.

Table 1 summarises baseline demographic and comorbidities by drug. Comorbidities appeared higher in those taking metformin. BMI and zBMI data were available for 91% (40/44) prescribed metformin and 89% (49/55) prescribed orlistat. All had BMI above the 98th centile (>2 SD). Prescriptions for metformin and orlistat increased with age, with orlistat largely prescribed to those aged 16 years or above.

**Table 1:** Demographics and comorbidities by drug

	Metformin	Orlistat
n (total, % GP-initiated)	44 (27%)	55 (89%)
Female (n, %)	40 (91%)	46 (84%)
BMI (mean kg/m^2^, SD)	35.9 (6.1)	37.6 (6.5)
zBMI (mean, SD)	3.2 (0.7)	3.2 (0.6)
Median age (range)	15.7 (6.5–19.2)	17.3 (13.8–18.8)
<12 years (n)	5	0
12–15.9 years (n)	19	9
16–17.9 years (n)	16	29
≥18 years (n)	4	17
Comorbidities (n,%)		
Hypertension	1 (2%)	0
Hyperinsulinism/insulin resistance	13 (30%)	0
Type 2 diabetes	0	3 (5%)
Dyslipidaemia	1 (2%)	3 (5%)
Emotional distress	12 (27%)	17 (31%)
Sleep apnoea	0	1 (2%)
Polycystic ovarian syndrome	32 (73%)	6 (11%)
Orthopaedic issues	3 (7%)	3 (5%)
Pervasive developmental disorder	3 (7%)	0
Hypothyroidism	1 (2%)	3 (5%)

BMI, body mass index;

### Drug initiation

**Figure 2:**
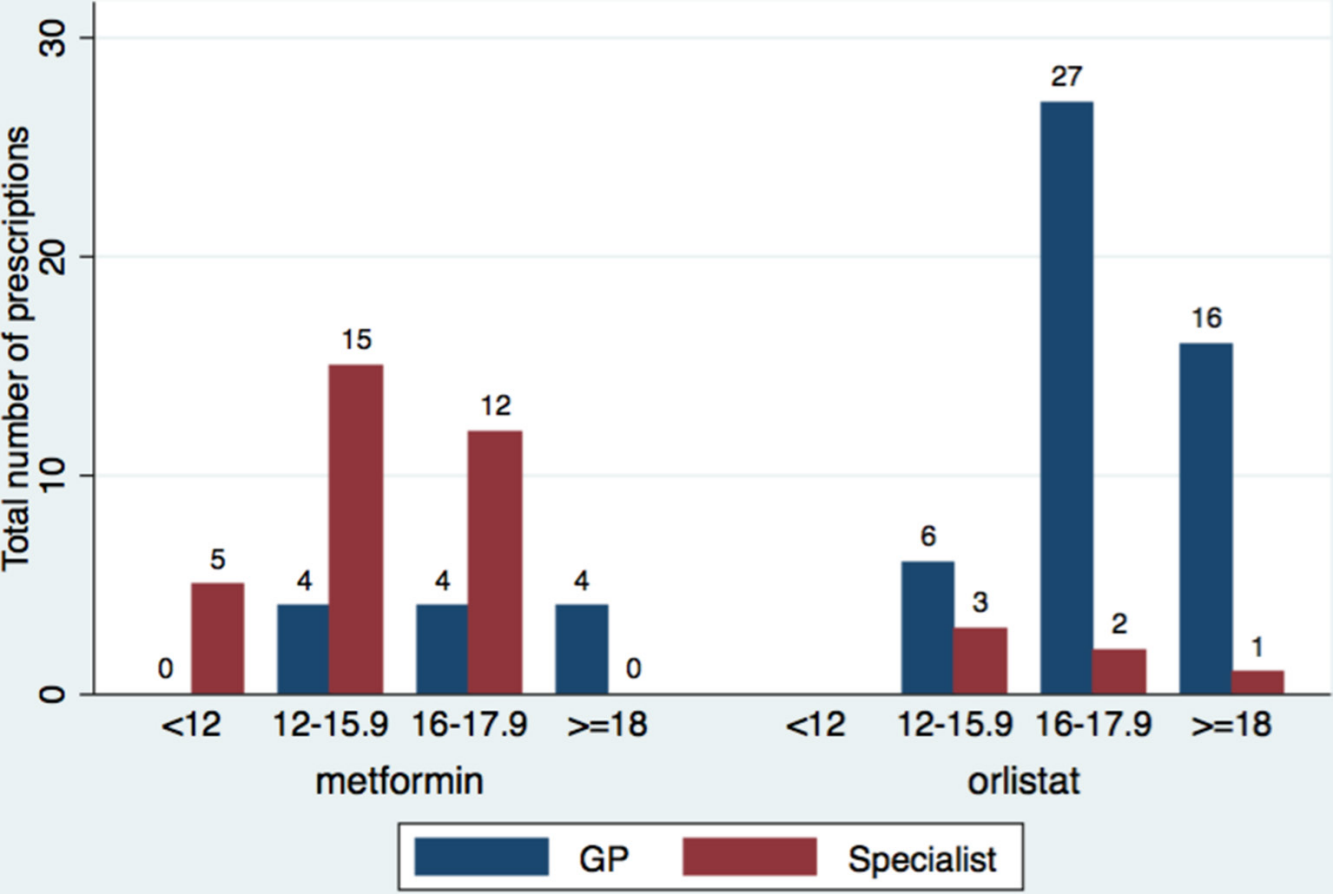
Bar graph summarising age at initiation by drug and initiator. GP, general practitioner.

Figure 2 summarises the frequency of drug prescription by age and drug initiator; 89% (49/55) of orlistat and 27% (12/44) metformin prescriptions were initiated in primary care independent of specialist advice. Orlistat was recommended by paediatricians (n=3), an adult physician, lipid clinic and dietitian, and metformin by paediatricians (n=19), gynaecologists (7), adult physicians (4) and endocrinologists (1).

Indications for metformin initiation were obesity together with (1) polycystic ovarian syndrome (70%, 31/44), (2) insulin resistance (25%, 11/44), (3) impaired glucose tolerance/impaired fasting glucose (9%, 4/44) and (4) obesity without known comorbidity (7%, 3/44).

### Drug monitoring

Medication monitoring in primary care was undertaken in 67% where initiated independently and 26% on specialist recommendation. GPs were made aware of adverse drug effects by two patients, both prescribed metformin; one had diarrhoea and the other nausea.

### Drug duration and termination

**Figure 3:**
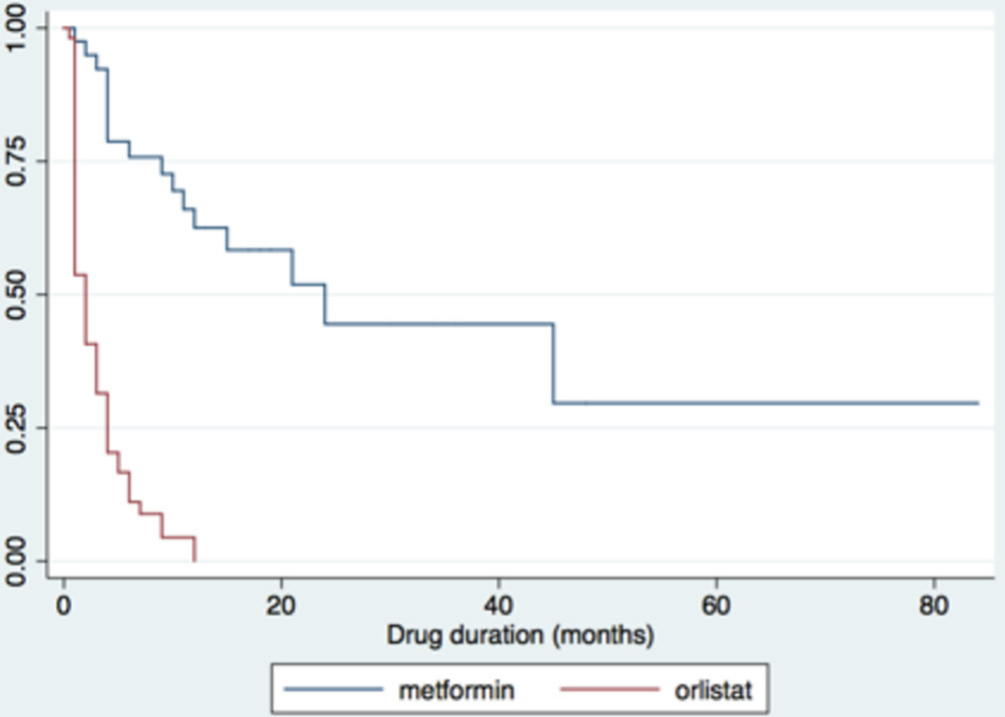
Kaplan-Meier survival curve demonstrating treatment duration of metformin and orlistat. Figure shows proportion still actively prescribed antiobesity drug for orlistat and metformin by time since initiation, beginning from time 0 (100% active prescriptions) out to 85 months (longer active prescription).

Duration of drug prescription is summarised in figure 3. The median supply of metformin was 10.5 months (IQR 4–18.5 months) compared with 2.0 months (1.0–4.0) for orlistat (p≤0.001). Over half of all metformin prescriptions (25/44) but only 5% of orlistat prescriptions (3/55) were active at the time of survey, defined as a new prescription issued within the preceding 3 months. There was a disparity between reported length of drug prescription and the amount of drug prescribed, suggesting non-continuous use at dose prescribed.

Twenty-seven patients had only a single prescription issued from primary care, being 45% (25/55) of all orlistat and 5% (2/44) metformin treatments. None of these single prescriptions were issued in the 3 months prior to the survey, and all were given a maximum of 1-month supply, making ongoing use highly unlikely.

The majority of all drug terminations were due to families not requesting repeat prescriptions (96% of orlistat and 89% metformin) rather than medically led terminations. GPs reported possible orlistat cessation in three cases due to lack of drug supply in pharmacies. Of four prescriptions actively terminated by a doctor (metformin=2, orlistat=2), two were due to lack of efficacy, one for lack of drug adherence, and the other two for reasons unknown.

### Adherence to NICE guidance

We restricted NICE compliance analysis to recommendations for children; 23 subjects were identified as definitely aged less than 18 years at drug initiation. All subjects were aged over 12 years (recommendation 1.8.4), and no participants were prescribed orlistat for over 12 months (1.9.11).

The following criteria were partially met: First, four (17%) were prescribed orlistat following specialist advice (1.8.5). Recommending specialists were paediatricians (n=3) and an adult physician, with one known to be part of a specialist multidisciplinary team (1.8.7). All prescriptions recommended by specialists were continued in primary care (1.8.8).

Second, comorbidities were reported in 57% of the sample (13/23) despite NICE requiring comorbidities to be present (1.8.6). These were emotional distress (7/23), hypothyroidism (3/23), type 2 diabetes (1/23), medulloblastoma (1/23), polycystic ovarian syndrome (1/23) or worsening of another chronic disease secondary to obesity (1/23). No patients had sleep apnoea. Low levels of comorbidity screening in primary care were reported, suggesting that higher number of comorbidities may have existed (6 of 23 were screened for psychosocial distress, 5 for hypertension, 2 each for type 2 diabetes and dyslipidaemia, and none for sleep apnoea).

No patients were prescribed a multivitamin (1.9.2). We did not assess screening for micronutrient intake or risk of vitamin deficiencies.

### Improving prescribing in primary care

**Figure 4:**
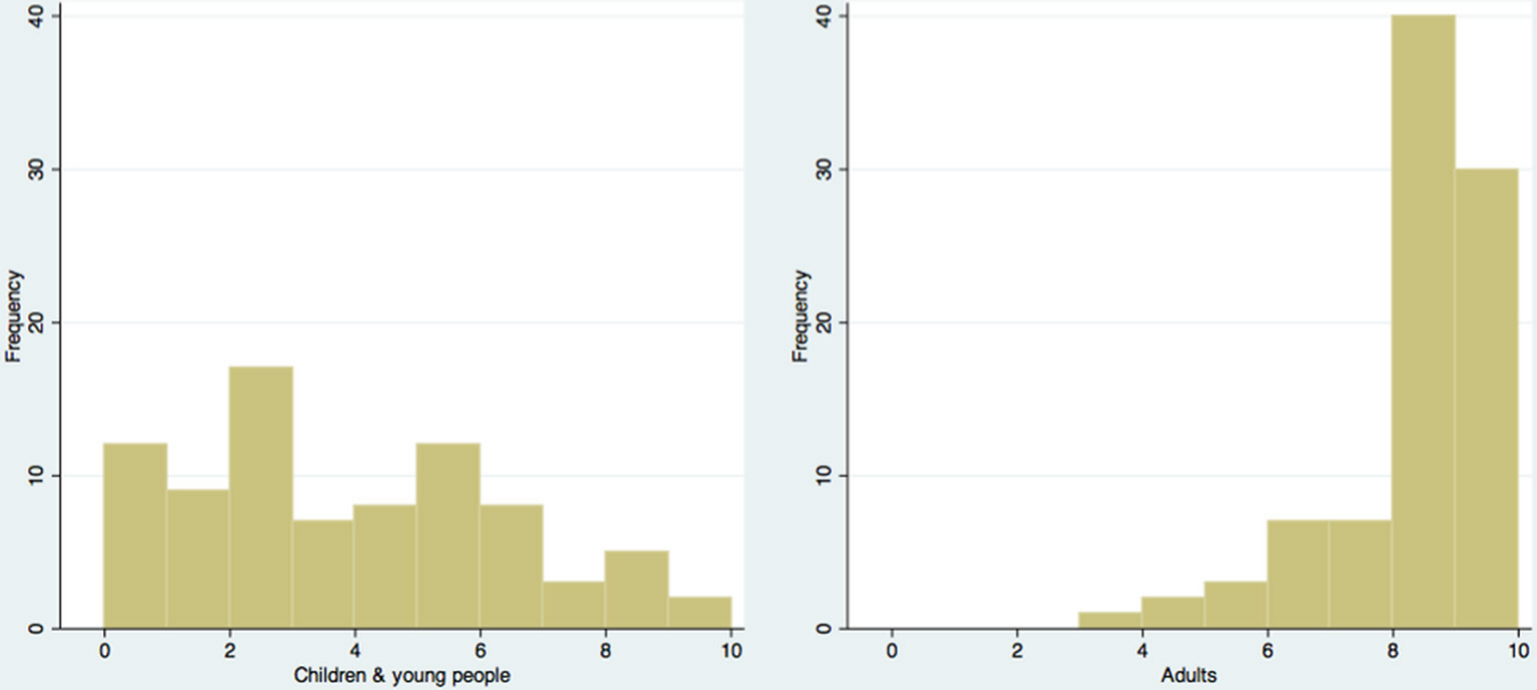
Histogram summarising general practitioner confidence in prescribing antiobesity drug to children and young people (left) and adults (right).

GP confidence in prescribing AOD to CYP and adults is summarised in figure 4 and shows a skewed inverse relationship. Confidence was higher for prescribing to adults (median=8, IQR 8–9) than children (3, 1–5) (p<0.001).

GPs used NICE guidance (orlistat n=20, metformin n=7), British National Formulary (orlistat 17, metformin 8), local prescribing guidelines (orlistat 8, metformin 11) and specialist guidance (orlistat 1, metformin 7) to support prescribing.

GPs perceived that 27% (n=12) of patients prescribed metformin and 13% (7) prescribed orlistat benefited from the drug, with half (50% metformin, 53% orlistat) reporting not knowing if there had been any benefits for the patient.

Thirty-five GPs provided brief free-text reflections of their experiences prescribing orlistat (n=20) and metformin(n=14). Three main themes arose. First, the use of metformin was mostly ascribed to polycystic ovarian syndrome rather than as a weight loss drug. One GP stated that (s)he “wouldn’t normally prescribe this just for weight loss.”

Second, there was controversy about whether AODs should be prescribed in primary care in this age group, with one saying (s)he “usually not prescribe for children” and another saying (s)he avoided orlistat “where possible.” Metformin was used either “on advice of specialist only,” or had specialist follow-up after initiation.

Third, GPs noted concern about the efficacy of these drugs. “Inadequate counseling,” lack of drug availability and patient compliance (“clearly patient was not able to comply”) were hypothesised reasons for ineffectiveness.

Sixty-two GPs wanted improved support, primarily split into two main themes. First, they requested improved age-related guidance for prescribing AOD that is ‘realistic’, with ‘clear [and] concise’ advice including ‘flow diagrams’ and ‘stepwise advice’. This would include instructions on assessment prior to initiation, indication indications, contraindications, monitoring, safety advice, duration, targets and indications for stopping treatment. Second, they wanted improved guidance for managing patients with obesity, namely advice about lifestyle management and details of available interventions. GPs requested details of “non-drug treatments,” including “community support for adolescents” and “special clinics for monitoring and support of patients.” One GP highlighted that “non-drug treatments need to be key alongside drug treatment.”

## Discussion

This is a detailed study of primary care prescribing of AODs in CYP at the individual patient level. Small numbers of prescriptions were issued in this age group, with most practices surveyed prescribing just a single AOD to a child or young person. However, clear patterns were detected that can help guide prescribing of current and future generations of AODs.

Recipients of an AOD were largely female, with two-thirds (65%) of the sample aged 16 or over. Two major prescribing patterns were seen: orlistat was largely initiated independently to those over 16 years, and metformin was largely recommended by specialists to girls with either polycystic ovarian syndrome or disturbances in glucose homeostasis. Comparison with NICE guidelines for orlistat showed low compliance with national prescribing recommendations, namely low prevalence of comorbidities and drug initiation without specialist advice. Given that most orlistat prescriptions were for those above 16 years, it could be hypothesised that those aged 16 years and over were treated as adults, with drugs prescribed in line with adult guidelines that do not necessitate presence of comorbidities.

Our findings augment the very limited existing data relating to AOD rates of initiation and cessation^14^ and experiences of CYP prescribed an AOD.^7^ A paired study by our research team investigating patient experiences of AODs found high levels of side effects, low levels of professional support managing these side effects, and ultimately families deciding to stop the AOD due to the disadvantages of the side effects outweighing the perceived benefits of the drugs.^7^ This contrasts with findings from this study where no patients actively discussed side effect profiles with their GPs, and GPs being aware of side effects in only two patients. This study does not explain the disconnect between the experiences of patients and clinicians, and further work should examine ways to support families so they are able to effectively manage the side effects of these drugs.

GPs reported low confidence in prescribing AOD to CYP, despite high levels of confidence when prescribing to adults. This fits with findings from our paired study, which showed that families notice this unease in primary care, and which can result in heightened familial concerns about AOD usage.^7^ GPs reported a desire for improved guidance on drug initiation and monitoring, and on lifestyle interventions, implying low overall confidence in managing childhood obesity. These findings suggest that current national guidelines are inadequate for the needs of primary care, and further work is needed to understand how GPs can better support those with obesity.

### Strengths and limitations

We used data from a nationally representative data set to identify patients prescribed an AOD with high rate of completion of questionnaires by GPs. Data collection relied on retrospective notes-based recall by GPs, increasing the likelihood of missing data. Individual item completion rates were variable, with some having only a few questions answered. We assumed that unanswered questions implied lack of evidence to support the questions. We were unable to ascertain the exact age of subjects, resulting in risk of misclassification bias, and we are likely to have underestimated the number of subjects who were prescribed orlistat under 18 years of age. We limited the scope of project, and as such we are unable to comment on variation in drug dosing. We were unable to evaluate non-participant bias due to lack of information about individual GP practices.

## Conclusions

Use of AOD including metformin in primary care is rare, particularly in men and those below 16 years. High rates of discontinuation were seen, primarily in those prescribed orlistat. Rates of compliance with NICE guidance for orlistat were low and GPs report low confidence in the use of AOD in this age group. Improved training and support for GPs is needed to guide AOD use in primary care, both for current and future generations of drugs.
