# Supplementary material for: Survey of antiobesity drug prescribing for obese children and young people in UK primary care
Source: BMJ Paediatr Open. 2017 Oct 25;1(1):e000104. doi: 10.1136/bmjpo-2017-000104 (PMC5862202; doi:10.1136/bmjpo-2017-000104)
Supplement: Supplementary file 1 [file bmjpo-2017-000104supp001.pdf]

GP Letter v2.0 17th February 2012

«AIS_Contact_Title» «AIS_Contact_Forename» «AIS_Contact_Surname»
«Practice_Name»
«Address1»
«Address2»
«Address3»
«Post_Code»

01/02/2012

Dear «AIS_Contact_Title» «AIS_Contact_Surname»

**Practice ID**	**Patient ID**	**Sex**	**Year of Birth**
**«Practice_ID»**	**«PatID»**	**«Sex»**	**«Year_of_Birth»**

Understanding Young People's Experiences of Anti-Obesity Drugs.

I am writing to invite you to participate in a study that researchers (University College London and The School of Pharmacy) are conducting using THIN data (the Health Improvement Network data). The study looks at **anti-obesity drug use in young people**. They are working with The Health Improvement Network (THIN) to recruit patients and the above patient has been identified as a potential participant.

We will aggregate any information that you send to us* with other patient related information that we have collected. Please be reassured that access to THIN data is only permitted for research protocols which have received prior approval by a Multi-Centre Research Ethics Committee (MREC). As you are already aware, all patient related information is, and remains, anonymised when it is included in this database and anonymised data are passed on to researchers and used by them in the strictest of confidence.

We have been working with the THIN database to recruit potential study participant and the above patient has been selected.

This project consists of two arms:

1. GP-completed questionnaire outlining prescribing practices. **We are asking you to complete the attached questionnaire.**
   Please be aware that an additional questionnaire needs to be completed if the patient is taking (or has been taking) several anti-obesity drugs (*Metformin, Orlistat, Sibutramine*): 1 questionnaire per drug.

2. Face to face interview with young person and family exploring their experiences using anti-obesity drugs. **We are asking you to send out the pre-prepared invitation pack to your patient and his/her parents/guardians.** If they choose to take part, researchers will be interviewing the young person and his/her parent/guardian and each interview will last approximately 45 minutes. This will take place in the participant's home, unless the participant chooses otherwise (further details in information sheets).

* Copyright and all other intellectual property rights or other rights used or embodied in or connected with the patient related information that we collect, or any part of such information, (to the extent that any such rights exist) including the manner in which it is presented, selected and/or arranged shall be and shall remain our property.

All data will remain confidential unless there are child protection concerns. If there are any child protection concerns, then the researcher will discuss these with you and his/her research team.

If your practice wishes to participate in the study please follow the next few steps.

- Complete the questionnaire(s) **(to be completed by GP).**

- If the patient is NOT eligible, please return your **completed questionnaire(s)** and the invitation pack to AIS (self addressed label enclosed).

- If the patient IS eligible, please return your **completed questionnaire(s)** to AIS and forward the enclosed invitation pack to patient.

The enclosed invitation pack to the patient consists of the following:

1. An invitation letter
2. Patient information sheets
3. Reply slip
4. A stamped addressed envelope

- Please insert **patient/carer's name and address** in the invitation letter. We would be grateful if you can send this letter on our behalf.

If there are questions from the patient that require an answer from us, please don't hesitate to get in touch.

We will pay you £00.00 via a BACS transfer per **completed questionnaire**. However, if for any reason you feel unable to participate in this study, please do not hesitate to contact me directly on 020 7554 0660 or email me on shobhna.koria@thin-uk.com**.**

Thank you for your continued support with our research.

Yours sincerely,

S. Koria

Shobhna Koria
AIS Research Manager

AIS Ref:

* Copyright and all other intellectual property rights or other rights used or embodied in or connected with the patient related information that we collect, or any part of such information, (to the extent that any such rights exist) including the manner in which it is presented, selected and/or arranged shall be and shall remain our property.

Questionnaire *(This should ideally be completed by the GP who issued the first prescription).*

Practice ID	Patient ID	Sex	Year of Birth

Anti-obesity medication:	*Metformin/Orlistat/Sibutramine*
Ethnicity	
Date of first prescription:	
Date of last prescription:	
Total supply issued (months):	

1. Has this patient been obese within the last 2 years?

☐ Yes **(Please return the completed questionnaire to AIS using the self addressed label enclosed and forward the invitation pack to the patient)**

☐ No: this patient is not eligible for this study. **(Please return the completed questionnaire & the invitation pack to AIS using the self addressed label enclosed)**

Please enter any measurements you have in the last 2 years (in any order)

Date								
Weight (kg)								
Height (cms)								

2. What co-morbidities has this patient had (tick all that apply)?

☐ Hypertension ☐ Hyperinsulinaemia ☐ Dyslipidaemia ☐ Type 2 diabetes ☐ Psychological dysfunction
☐ Psycho-social distress (e.g. low self-esteem, teasing and bullying)
☐ Mental health (e.g. depression, eating disorder) ☐ Sleep apnoea
☐ Weight-related exacerbations of conditions such as asthma ☐ Polycystic ovarian syndrome
☐ Orthopaedic/mobility issues related to weight (please specify**):___________________________________**
☐ Other (please specify):**____**

3. How was this medication initiated?

☐ GP issued this medication without secondary/tertiary care advice (go to question **X**)
☐ GP issued this medication after advice from secondary/tertiary care team (go to question **Y**)

X) Which of the following did the patient receive BEFORE initiation of medication (by any member of team, e.g. dietician, councillor, practice nurse)

A. General assessement:

☐ Dietetic review ☐ Lifestyle review ☐ Medical causes of obesity
☐ Growth and pubertal status ☐ Family history of obesity and co-morbidities
☐ Records inadequate to answer

B. Assessment of co-morbidities:

☐ Hypertension ☐ Hyperinsulinaemia ☐ Dyslipidaemia ☐ Type 2 diabetes
☐ Sleep apnoea ☐ Exacerbations of conditions such as asthma
☐ Psycho-social distress (e.g. low self-esteem, teasing, bullying) ☐ Mental health (e.g. depression, eating disorder) ☐ Records inadequate to answer

C. Motivation Review:

☐ Review of willingness and motivation to change
☐ Records inadequate to answer

D. Other treatment options attempted:

☐ Mental/emotional health support ☐ Exercise prescription
☐ Structured community intervention (e.g. MEND) ☐ Records inadequate to answer

Y) If treatment was recommended by secondary/tertiary care:

A. Who recommended starting this medication?

☐ Paediatrician ☐ Adult physician ☐ Other (please specify):**_________________________**

AIS Reference:

B. Was this practitioner part of a multi-disciplinary team with expertise in managing obesity in this age group?

☐ Yes ☐ No ☐ Don't know

C. Did patient require support from primary care with this medication?

☐ Yes-Side-effect ☐Yes – Efficacy ☐ Yes- other(please specify):________________

☐No ☐ Don't know

4. What is the current status of this medication?

☐ New prescription issued within last 3 months

☐ Patient stopped taking / not requested prescription for more than 3 months.

Please specify reasons if known: ____

☐ Medication stopped by doctor. Why (tick all that apply):

☐ Lack of efficacy ☐ Non-concordance ☐ Adverse effects

5. Were any nutritional/vitamin supplement prescribed?

☐ No ☐ Yes, please specify: ____

6. Who reviewed the patient to assess effectiveness, adverse effects and adherence (tick all that apply)

☐ GP ☐ Paediatrician ☐ Adult physician ☐ Other (please specify):________________ ☐ Don't know

7. Were there any adverse effects of this drug?

☐ No ☐ Yes, please specify: ______________________________ ☐ Don't know

8. Did the patient's weight change while on the medication?

☐ Loss, how much (if known or approx.):______________________ ☐ Neutral

☐ Gain, (if known or approx.):______________________________ ☐ Don't know

9. Do you think this medication benefitted the patient

☐ Yes ☐No ☐Unsure

10. Metformin only: What was the indication for prescribing metformin (tick all that apply)

☐ Diabetes ☐ Polycystic ovarian syndrome ☐ Insulin resistance / hyperinsulinism

☐ Impaired glucose tolerance / Impaired fasting glucose ☐ Obesity with none of the above

☐ Other: please specify: ____

11. What tools were used to support the prescribing of this medication?

☐ NICE guidance ☐ MIMS ☐ BNF ☐ GP notebook ☐ Local prescribing recommendations

☐ Other: please specify:____

12. How confident do you feel about prescribing anti-obesity medications, using a scale 1-10, (10 = very confident)

To adults: ____________________________

To children (<18 years):__________________

13. Any comments regarding prescribing of this drug:

____

____

14. We will be developing a guide to support clinicians prescribing anti-obesity drugs. What would you like to see in this guide:

____

____

This patient is eligible and I have forwarded the invitation pack to the patient ▯ **YES** ▯ **NO**

Please tick one box as appropriate

AIS Reference:

Cegedim Strategic Data (THIN)

Additional Information Services from THIN, 1 Canal Side Studios, 8-14 St Pancras Way, London NW1 0QG

Registered Number 04396589 **Telephone**: +44 (0)20 7388 8215 **Facsimile**: +44 (0)20 7388 8492
